# Supplementary material for: Graphene Oxide-Based Memristive Logic-in-Memory Circuit Enabling Normally-Off Computing
Source: Nanomaterials (Basel). 2023 Feb 13;13(4):710. doi: 10.3390/nano13040710 (PMC9963271; doi:10.3390/nano13040710)
Supplement: Supplementary file 1 [file nanomaterials-13-00710-s001.zip › nanomaterials-2199400-supplementary.pdf]

nanomaterials

MDPI

Supplementary Materials

Graphene Oxide-Based Memristive Logic-in-Memory Circuit Enabling Normally-Off Computing

Yeongkwon Kim [1], Seung-Bae Jeon [2,*] and Byung Chul Jang [1,3,*]

[1] School of Electronic and Electrical Engineering, Kyungpook National University, 80 Daehakro, Bukgu, Daegu 41566, Republic of Korea
[2] Department of Electronic Engineering, Hanbat National University, 125 Dongseo-daero, Yuseong-gu, Daejeon 34158, Republic of Korea
[3] School of Electronics Engineering, Kyungpook National University, 80 Daehakro, Bukgu, Daegu 41566, Republic of Korea
* Correspondence: sbjeon@hanbat.ac.kr (S.-B.J.); bc.jang@knu.ac.kr (B.C.J.)

1. HRS/LRS Resistance and V_{SET}/V_{RESET} Distributions in Terms of Device-to-Device

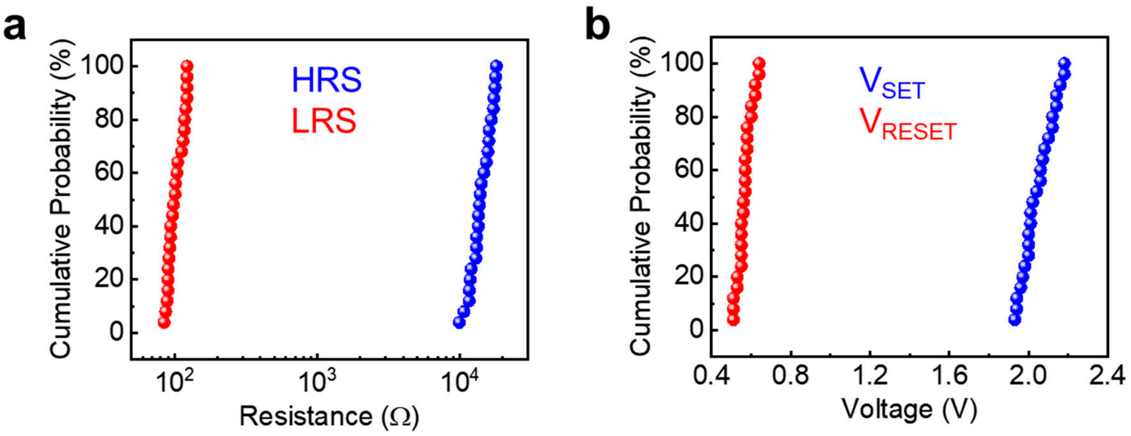

Figure S1. Device-to-device distribution of (**a**) HRS/LRS resistance and (**b**) V_{SET} and V_{RESET} for GO-based memristor 5 × 5 array.

2. Benchmarking Table with Memristive Logic-in-Memory Circuit

Table S1. Comparision of the previously reported memristive logic-in-memory circuit device.

	Material	On/Off raito	V_{SET}/ I_{SET}	V_{RESET}/ I_{RESET}	Endurance	Retention	Logic gate
pEGDMA-memristor [42]	Polymer	> 200	1.5 V	-1 V	500 cycles	> 10^7 sec	NOT, NOR, OR, NAND, AND
pV3D3-memrsitor [15]	Polymer	> 10^7	3 V	0.5 V	500 cycles	10^5 sec	NOT, NOR, OR, NAND, AND
Ta_2O_5-memristor [20]	Inorganic	100	−1 V	2 V	N.A.	N.A.	NIMP, OR, XOR
Bi_2O_2Se-transistor [19]	2D Material	> 100	3 V	0.5 V	100 cycles	> 1600 sec	NAND
STT-MRAM [43]	Inorganic	> 2	91 μA	134 μA	N.A.	N.A.	NOR
TaOx-memristor [44]	Inorganic	Inorganic	0.7 V	−1.5 V	N.A.	N.A.	IMP, NIMP, NAND, AND, OR, NOT
This work	2D Material	> 100	0.5 V	2V	300 cycles	10^4 sec	NOT, OR, OR, NAND, AND
